# Antibiotic Innovation May Contribute to Slowing the Dissemination of Multiresistant *Streptococcus pneumoniae*: The Example of Ketolides

**DOI:** 10.1371/journal.pone.0002089

**Published:** 2008-05-07

**Authors:** Lulla Opatowski, Laura Temime, Emmanuelle Varon, Roland Leclerc, Henri Drugeon, Pierre-Yves Boëlle, Didier Guillemot

**Affiliations:** 1 Unité de Pharmacoépidémiologie et Maladies Infectieuses, Institut Pasteur, Paris, France; 2 U657, INSERM, Paris, France; 3 Chaire Hygiène & Sécurité, Conservatoire National des Arts et Métiers, Paris, France; 4 Centre National de Référence des Pneumocoques, Assistance Publique des Hôpitaux de Paris, Hôpital Européen Georges Pompidou, Paris, France; 5 EA 2128, Interactions hôte et microorganismes des épithéliums, Université de Caen and Service de Microbiologie, CHU Côte de Nacre, Caen, France; 6 Laboratoire de Bactériologie, Virologie, Hygiène Hospitalière, Hôpital G&R Laënnec, Nantes, France; 7 U707, INSERM, Paris, France; 8 UPMC-Paris6, UMR S 707, Paris, France; 9 Hôpital Saint-Antoine, Assistance Publique des Hôpitaux de Paris, Paris, France; 10 Université de Versailles Saint Quentin, Versailles, France; 11 Unité de Santé Publique, Hôpital Raymond Poincaré, Assistance Publique des Hôpitaux de Paris, Paris, France; London School of Hygiene and Tropical Medicine, Peru

## Abstract

**Background:**

Despite increasingly frequent bacterial resistance to antibiotics, antibacterial innovation is rare. Ketolides constitute one of the very few new antibiotic classes active against *Streptococcus pneumoniae* developed during the last 25 years. Their mechanism of action resembles that of macrolides, but they are unaffected by common resistance mechanisms. However, cross-resistance to ketolides has been observed in some macrolide-resistant strains. We examined how new antibiotic exposure may affect overall pneumococcal resistance patterns in the population. The aims of this study were to assess the potential dissemination of newly emerged resistances and to control the selection of strains already multiresistant to existing antimicrobials.

**Methodology/Principal Findings:**

We developed an age-structured population model for *S. pneumoniae* transmission in a human community exposed to heptavalent vaccine, and *β*-lactams, macrolides and ketolides. The dynamics of intra-individual selection of resistant strains under antibiotic exposure and interindividual transmission were simulated, with antibiotic-specific resistance mechanisms defining the path to co-resistances and cross-resistances, and parameters concerning the French situation. Results of this simulation study suggest that new antibiotic consumption could markedly slow the diffusion of multiresistant strains. Wider use was associated with slower progression of multiresistance. When ketolides were prescribed to all ages, resistance to them reached 10% after >15 years, while it took >40 years when they were prescribed only to adults. In the scenario according to which new antibiotics totally replaced former antimicrobials, the *β*-lactam resistance rate was limited at 70%.

**Conclusions:**

In a context of widespread vaccination and rational use of antibiotics, innovative antibiotic, prescribed to all age groups, may have an added impact on multiresistant-strain dissemination in the population.

## Introduction

Bacterial resistance to antibiotics has become a major public health problem worldwide and direct consequences are now being documented in terms of increased morbidity, mortality and hospitalization costs [1]–[4]. Minimizing selection of pathogenic bacteria that have acquired mechanisms of resistance to antibiotics has become a top priority of international public health authorities. *Streptococcus pneumoniae* is a major cause of morbidity and mortality worldwide [5], and the main cause of community-acquired respiratory tract infections, e.g., otitis media, sinusitis and pneumonia. It is also a major pathogen responsible for invasive disease, like septicemia and meningitis.

In recent decades, antibiotic-resistant and multiresistant *S. pneumoniae* strains have spread throughout the community. In some European countries, such as France or Spain, penicillin non- susceptible strains represent >50% of all pneumococci, as do macrolide-resistant strains [6]. Multiresistance is also being observed more and more frequently. Despite this increased antibiotic resistance, very few new antibacterial classes have been developed by pharmaceutical companies over the last few decades [7].

The 7-valent pneumococcal conjugate vaccine (PCV7) markedly decreased resistant *S. pneumoniae* infections [8], [9], but recent observations suggest the emergence of non-vaccine serotypes with high levels of resistance to all approved antibiotics [10].

Since antibiotic exposure is the main driving force behind the selection of strains harboring resistance to current drugs, a new class of antibiotic, without preexisting resistant strains, could help control the selection of resistance to older drugs, but emergence of new resistances to these new antibiotics is to be expected. The aims of this study were to examine that hypothesis using mathematical modeling, and to optimize strategies of new compound use by maximizing the time until emergence of new resistances and controlling the selection of resistant strains to preexisting antimicrobials.

This investigation was specifically conducted on ketolides, one of the newest antibiotic classes marketed for community-acquired respiratory tract infections.

## Materials and Methods

We built a compartmental model of the transmission of *S. pneumoniae* in the French community under multiple antibiotic exposures. The model was specific to different mechanisms of resistance and the natural history of pneumococcal colonization. Using this model, we explored the diffusion of colonizing resistant and multiresistant pneumococci, according to antibiotic exposure. The objectives were to anticipate trends in new ketolide-resistance selection and multiresistance selection. First, we describe the model structure, then detail the parameters and, finally, present the scenarios that were simulated.

### Model Description

The model developed herein takes into account 3 different antimicrobial classes (P: penicillin as an example of *β*-lactams, M: macrolides and K: ketolides), PCV7, and pertinent parameters concerning the French situation taken from the literature (Table 1).

**Table 1:** List of model parameters and their values.

Parameter	Variable	Mean value
Birth rate	*μ*_0_	0.000250 week^−1^
Death rate of		
Young children (<2 years)	*μ*_1_	0.000112 week^−1^
Older children (2–15 years)	*μ*_2_	0.000047 week^−1^
Adults (>15 years)	*μ*_3_	0.000295 week^−1^
Infectious contact rate within		
Young children	*β*_11_	0.9 week^−1^person^−1^
Older children	*β*_22_	0.7 week^−1^ person^−1^
Adults	*β*_33_	0.3 week^−1^ person^−1^
Infectious contact rate between		
Young and older children	*β*_12_	0.68 week^−1^ person^−1^
Young children and adults	*β*_13_	0.68 week^−1^ person^−1^
Older children and adults	*β*_23_	0.68 week^−1^ person^−1^
Frequency of antibiotic exposure in 2002 among		
Young children	[*α*_00_, *α*_10_]	[0.046, 0.0065] week^−1^
Older children	[*α*_01_, *α*_11_]	[0.018, 0.0042] week^−1^
Adults	[*α*_02_, *α*_12_]	[0.0086, 0.0044] week^−1^
Vaccination rate of young children (<2 years)	*v*	30% (2004)–90% (2007)
Duration of vaccine immunity	*d_v_*	13 years
Probability of non-decolonization after 4 days of		
Penicillin for carriers of S_P_, I_P_ and R_P_ pneumococci	[*σ*_0_, *σ*_1_, *σ*_2_]	[0.05, 0.35, 0.6]
Macrolide for carriers of S_M_ and R_M_ pneumococci	[*σ*_3_, *σ*_4_]	[0.03, 0.62]
Ketolide for carriers of S_K_ and R_K_ pneumococci	[*σ*_5_, *σ*_6_]	[0.03, 0.62]
Probability of incremental resistance of		
*β*-Lactams, S→I, S→R and I→R	[*p*_0_, *p*_1_, *p*_2_]	[0.0125, 1.5×10^−6^, 5×10^−5^]
Macrolides, S→R	*p*_3_	0.011
Ketolides, S→R	*p*_4_	0.01
Time until treatment acts	1/*ν*	4 days
Duration of antibiotic exposure	1/*γ*	8 days
Duration of colonization of		
Young children	1/*λ*_1_	9 weeks
Older children	1/*λ*_2_	4 weeks
Adults	1/*λ*_3_	2 weeks

**NOTE.** S, susceptible; I, intermediate; R, resistant.

**Figure 1:**
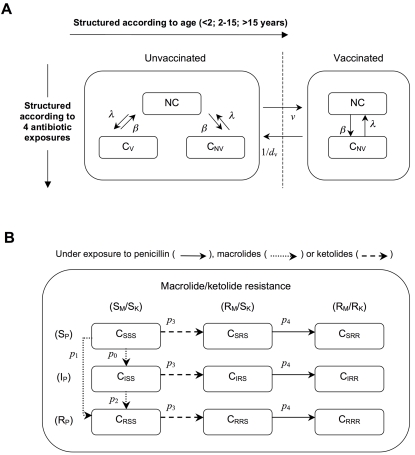
Schematized model structure. The model is structured according to age and antibiotic exposure: 3 age groups (<2, 2–15, >15 years) and 4 types of exposure (none, penicillin, macrolides, ketolides). (*A*) For each age/antibiotic exposure combination, 3 possible carriage (C) statuses are described: absence of carriage (NC) and carriage of either the vaccine-type (C_V_) or non-vaccine-type (C_NV_) pneumococcal strains. For vaccinated individuals, only 2 carriage statuses exist: NC and C_NV_. (*B*) Model substructure. More precisely, colonized individuals are divided into 9 subcompartments corresponding to carriage of a pneumococcal strain with one of the 9 possible levels of resistance, ranging from susceptible to the 3 antibiotic classes (SSS) to resistant to all of them (RRR), and all possible susceptibility-level combinations in-between. The subscripts correspond to susceptible (S), intermediate (I) or resistant (R) to *β*-lactams (P), macrolides (M) and ketolides (K). Because of the cross-resistance hypothesis, ketolide-resistant strains are always resistant to macrolides.

To reproduce the selection and spread of resistant bacteria in the community through interindividual transmission of *S. pneumoniae* strains, the population was divided into several groups or compartments. Compartments were structured with respect to age, vaccination status, colonization and antibiotic exposure (Figure 1). To take into account differences according to the hosts' ages, the population was divided into 3 age classes corresponding to young children (0–2 years), older children (2–15 years) and adults (>15 years). Hosts entered the population at birth as young children non-carriers. Mortality rates were defined for each class. We assumed that a fraction of young children was vaccinated each year. Vaccinated individuals were protected against carriage of serotypes included in PCV7, but not against carriage of serotypes not included in it.

Independently of their carriage status, individuals could be exposed to *β*-lactams (penicillin) and macrolides, as they are the main antibiotic classes prescribed in France [11]; and ketolides as the antibiotic innovation. We assumed that individuals were exposed to only 1 antibiotic class at a time. Because prescription frequencies depend on age and antibiotic classes, the matrix *α*[*i,j*] describes the rates of exposure as a function of age *i* and antibiotic class *j*. Under antibiotic exposure, strains with higher levels of resistance can emerge through genetic events and be selected, thereby replacing the strains originally harbored in the host in whom colonization had not been eliminated.

### Parameters

Parameter values are listed in table 1. We used demographic data from the French National Institute of Statistics and Economical Studies (INSEE) [12] for the birth rate (*μ_0_*) and mortality rates of each age class (*μ*_1_, *μ*_2_, *μ*_3_). Pneumococcal colonization was defined as decreasing with age [13], [14]: 50% in young children (<2 years old), 30% in older children (2–15 years old) and 10% in adults (>15 years old). The contact matrix was considered to be symmetric. Values of the age-specific contact rates *β*[*i,j*] were numerically calibrated so that the prevalence of overall carriage at the model equilibrium corresponded to that observed in the French population.

In the absence of antibiotic exposure, natural immunity was defined as inducing carriage clearance in colonized individuals, according to their age: on average, 2 months for young children (*1/λ*_1_), 1 month for older children (*1/λ*_2_) and 2 weeks for adults (*1/λ*_3_) [15]–[18].

#### Pneumococcal conjugate vaccine

PCV7 protects against both invasive disease and pharyngeal colonization [8], and has been recommended for young children in several countries [19]–[21]. In France, about 30% of children <2 years old, corresponding to all children in day care, were vaccinated each year before 2006. In 2006, recommendations were extended to systematic vaccination of all children <2 years old [22]. Previously developed models, describing the effect of vaccination on pneumococcal carriage and resistance, predicted replacement of serotypes included in the vaccine by non-vaccine serotypes [23], [24]. In our model, young children were vaccinated at rate *v*. Vaccinated children were assumed to have received 4 doses of PCV7 (including 1 booster dose), and thus to be fully immunized against serotypes included in PCV7. We assumed progressively increasing vaccination coverage between 2002 and 2004 from no-vaccination to vaccination of 30% of young children. After 2007, date of systematic vaccination of young children, we considered young children's vaccine coverage to be 90%. Vaccine immunity (*d_v_*) was supposed to last throughout childhood.

#### Antibiotic-exposure parameters

We considered the global antibiotic consumption regardless of indication of the drugs. We used annual global data published by the French National Health Insurance Agencies (CNAM–TS, CANAM) [25], covering >95% of the population, to evaluate prescription frequencies for the 3 antibiotic classes of interest for each age class. Consumption varied with age: children <5 years old had high rates of exposure, while prescriptions were quite low for adults. To be consistent with the objective of overall antibiotic-use reduction in France, a progressive and homogeneous decrease of global antibiotic exposure was assumed for all age groups (reaching 25%, 20% and 10% reductions over a 5-year period in young children, older children and adults, respectively). We investigated scenarios of ketolide exposure from the current status to their total replacement of old antibiotic classes, as developed below in detail.

Treatment duration (1/*γ*) was assumed to be the same regardless of the antibiotic prescribed (8 days). We considered that the antibiotic effect on colonization (*σ*) started after an average of 4 days of exposure (1/*ν*) and that it depended on the colonizing strain's level of susceptibility (see below).

#### Antibiotic resistance

Three stages of *S. pneumoniae* penicillin-susceptibility, corresponding to increasing minimum inhibitory concentrations (MIC), were modeled [26]: susceptible (S_P_), intermediate (I_P_), and resistant (R_P_), for strains with respective MIC<0.125, 0.125≤MIC<2 and MIC>2 mg/mL. Furthermore, since *S. pneumoniae*'s macrolide-resistance acquisition by ribosomal structure modification can be viewed as an on/off mechanism [27], corresponding to resistant/susceptible bacteria, macrolide-susceptible and -resistant (S_M_ and R_M_) strains were considered. Ketolides have a mechanism of action similar to that of macrolides [28], but R_M_ strains are generally susceptible to ketolides (S_K_).

Importantly, the few documented examples of ketolide-resistant (R_K_) strains concerned those already resistant to macrolides. That observation suggests that ketolide resistance is conditioned by previous resistance to macrolides (cross-resistance) [29]–[31]. Therefore, macrolide resistance was considered to be a prerequisite for the later emergence of ketolide resistance, which was ascribed to all strains already R_M_. Consequently, all R_K_ strains colonizing individuals in the model were also R_M_.

Emergence of resistance or an increase of its level was possible in colonized individuals exposed to antibiotics. Resistance levels evolved independently for penicillin and macrolides. Their rates of increased resistance (S_P_→I_P_, S_P_→R_P_, I_P_→R_P_ and S_M_→R_M_) were numerically estimated so that simulation results agreed with the observed historical trends (before 1992) of emergence and diffusion of R_P_ and R_M_ strains (*p_i_*). For ketolides, the same rates as those estimated for macrolides were used.

#### Multiresistance

Because pneumococcal strains can combine resistances to different antibiotics, 2 types of multiresistant strains were included and evaluated in the model: penicillin–macrolide-resistant strains (denoted: (I_P_/R_P_)+R_M_) and penicillin–macrolide–ketolide-resistant strains ((I_P_/R_P_)+R_M_+R_K_). Macrolide–ketolide-resistant (R_M_+R_K_) but penicillin-susceptible (S_P_) strains were not considered multiresistant strains, because of the macrolide–ketolide cross-resistance.

### Model Fitting and Simulations

To validate model predictions, we used historical data on antibiotic consumption in France [32], and compared simulation results with pneumococcal resistance and multiresistance rates from 1992 to 1997 reported by the French National Center for Pneumococci (CNRP), which coordinates the national surveillance system of pneumococcal infections [33].

#### Initialization

We initialized the model with a population in which resistant pneumococcal rates were equivalent to the 2002 French situation [33] with >50% of I_P_/R_P_ strains, >55% of R_M_ strains and 0.1% preexisting R_K_ strains [34]–[37].

#### Scenarios

**Figure 2:**
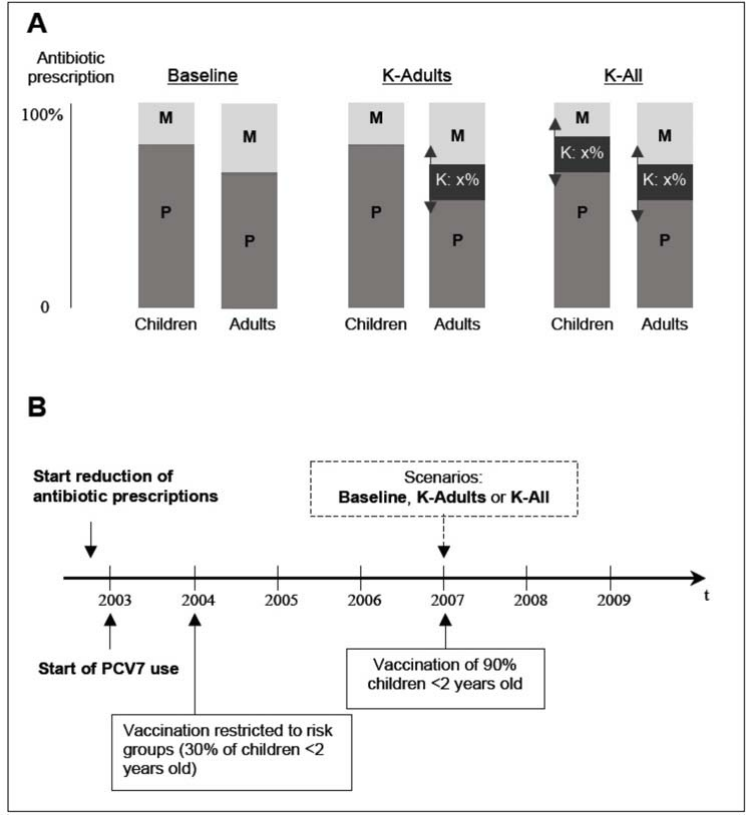
Description of epidemiological pertinent facts and scenarios, showing how parameters of antibiotic-exposure rates (P, penicillin; M, macrolides; K, ketolides) and PCV7 immunization rates evolved in the simulations. (*A*) Scenarios of ketolide introduction. Baseline: K are never prescribed; K-Adults: K are prescribed only to adults, to replace P and M; K-All: K are prescribed to all individuals, to replace P and M. In the two K-scenarios, the level of replacement varies from 0% to 100%. (*B*) Timeline of relevant epidemiological changes included in the model.

We investigated the impact of ketolide exposure by simulating 3 scenarios (Figure 2). The first, Baseline, corresponded to no ketolide-use, i.e. the French situation before 2005. The second scenario, K-Adults, consisted of replacing 25%, 50%, 75% or 100% of adult prescriptions with ketolides. The last scenario, K-All, consisted of replacing 25%, 50%, 75% or 100% of prescriptions with ketolides in all age groups.

To study R_K_ emergence and selection, we estimated the times required for 1% or 10% of strains colonizing the population to become R_K_. Furthermore, we simulated the expected dynamics of multiresistance diffusion over the next 50 years, defined as resistance to at least penicillin and macrolides ((I_P_/R_P_)+R_M_), according to the scenarios.

#### Sensitivity analysis

A sensitivity analysis was undertaken to evaluate the dependence of model predictions (percentages of R_K_ and multiresistant strains in the colonized population) on small variations of all input parameters (such as the contact matrix, antibiotic exposure levels, etc.). The parameters were allowed to vary within a range of their possible values. Latin Hypercube Sampling (LHS) was used and partial rank correlation coefficients (PRCC) were computed for all parameters [38].

## Results

**Figure 3:**
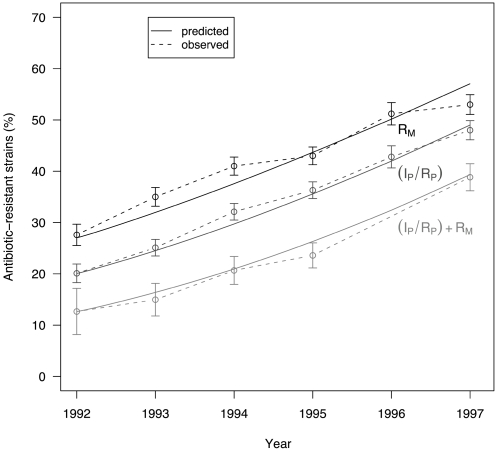
Dynamics of antibiotic-resistant and -multiresistant *S. pneumoniae* strains, 1992–1997: observed (dashed line) and predicted (black line) percentages. R_M_ and I_P_/R_P_ lines correspond, respectively, to macrolide and *β*-lactam–resistance rates. (I_P_/R_P_)+R_M_ represents percentages of *β*-lactam- and macrolide-multiresistant *S. pneumoniae* strains.

The historical time course of macrolide resistance, penicillin resistance and penicillin-macrolide multiresistance from 1992 to 1997 were well reproduced by model simulations (Figure 3). In a previous study using data from the United States, we showed that the simulated decrease over the first 3 years of mass vaccination was consistent in magnitude with the observed data, in terms of invasive disease incidence and in terms of R_P_ [24].

### 

#### Emergence of ketolide-resistant strains

**Figure 4:**
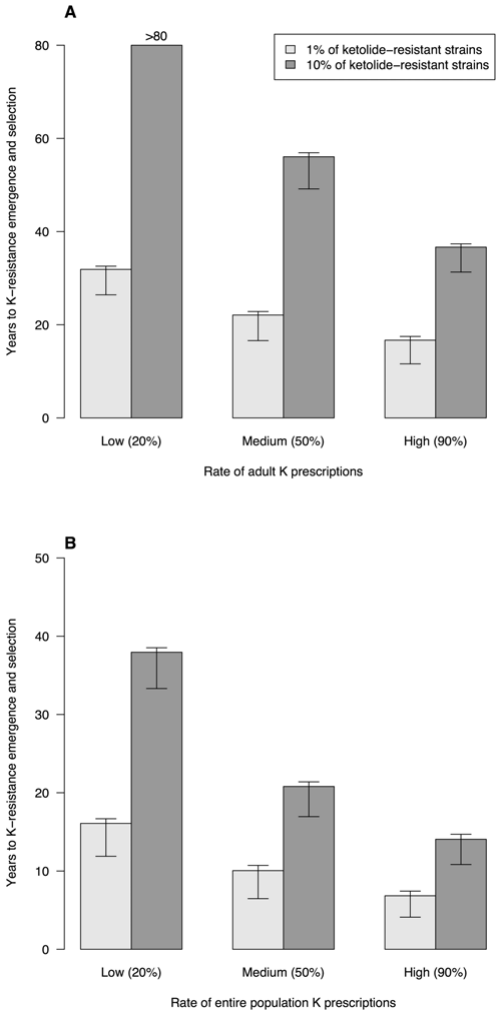
Time to emergence of ketolide-resistant *S. pneumoniae*. Times to reach 1% or 10% resistant strains among colonized individuals as a function of the rate of ketolide prescriptions replacing penicillin and macrolides in adults (*A*) or the entire population (*B*) are shown. Three ketolide-prescription levels are depicted: low, medium and high replacement rates of total antibiotic consumption. For these simulations, times were computed using *p*_4_ = 0.01 as the probability of ketolide-resistance emergence in strains. The T-bars represent the lower and upper prediction intervals, corresponding to *p*_4_ = 0.1 and 0.001, respectively.

In scenario K-Adults, ketolides were prescribed only to adults. Times to emergence of 1% and 10% R_K_ pneumococcal strains in the population are presented in figure 4A for different levels of ketolide exposure. For up to 50% of ketolide use among adults, the mean emergence time of 10% R_K_ exceeded 50 years. Under the hypothesis that ketolides effectively replaced high rate of the preexisting antimicrobials in total adult antibiotic consumption, it still took >35 years to reach 10% R_K_.

When ketolides were prescribed to all ages (scenario K-All), times before R_K_ emergence were shorter (Figure 4B). When ketolides replaced at least 20% of all preexisting antibiotic consumption, it took <40 years to reach 10% R_K_, whereas for quasi-total (90%) ketolide replacement of former antibiotics, simulations predicted that 10% R_K_ could be reached within a dozen years.

#### Dynamics of multiresistant strains

**Figure 5:**
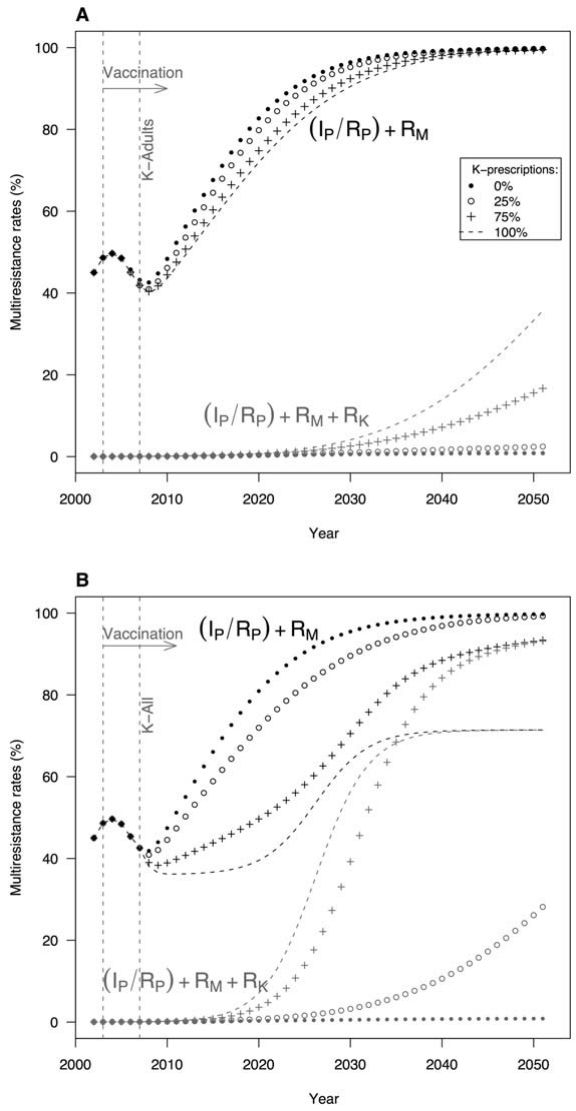
Impact of ketolide prescriptions on the emergence and selection of multiresistant *S. pneumoniae*. Evolutions of multiresistant-strain carriage as a function of time are shown. These simulations were carried out according to the 2 scenarios of K prescriptions: (*A*) K-Adults: ketolides were only used by adults after 2007, with 4 different replacement rates of adult antibiotic prescriptions: 0% (•); 25% (o); 75% (+); and 100% (−). (*B*) K-All: K prescriptions were extended to the entire population (including children) with the same replacement rates as previously.

When the new drugs were prescribed only to adults (scenario K-Adults, figure 5A), the dissemination of (I_P_/R_P_)+R_M_ multiresistant strains was barely slowed by ketolide use over the first few years. However, after R_K_ emergence and selection, the diffusion of strains resistant to P, M and K ((I_P_/R_P_)+R_M_+R_K_) was accelerated with increased ketolide use, as a consequence of the co-selection of R_M_+R_K_ strains under ketolide exposure, owing to macrolide–ketolide cross-resistance. Twenty years after R_K_ emergence and its selection in the population, no significant differences in the frequencies of carriage of multiresistant pneumococcal strains remained between the scenario using ketolides (K-Adults) and the one not using them (Baseline).

When ketolide prescriptions were extended to the entire population (scenario K-All, figure 5B), the dissemination of (I_P_/R_P_)+R_M_ strains differed markedly according to the prescription level. The higher the ketolide-prescription rate, the lower the initial diffusion of (I_P_/R_P_)+R_M_ strains, but the emergence of (I_P_/R_P_)+R_M_+R_K_ multiresistant strains no longer slowed the resistance rise and even enhanced it. K-All (I_P_/R_P_)+R_M_+R_K_ multiresistance appeared more rapidly than in K-Adults, and quickly encompassed all resistant strains. Importantly, when ketolides replaced >50% of current antibiotics, the simulations showed 2-step dynamics: first, a strong deceleration of (I_P_/R_P_)+R_M_ strain dissemination; second, an acceleration, increasing with the rise of the replacement rate. In the extreme case in which ketolides totally replaced current antibiotics (100%), the maximum multiresistance level was reached after only 20 years. It should be noted that, when ketolides totally replaced preexisting antibiotics, the model predicted that multiresistant-strain diffusion would cease at ∼70%, maintaining a reservoir of S_P_ strains.

#### Sensitivity analysis

The most significantly sensitive parameters are listed in table 2. Concerning R_K_ and multiresistance rate outcomes, the model was particularly sensitive to the frequencies and duration of antibiotic exposure: when either increased, the proportion of resistant strains increased. Moreover, the model was sensitive to the average time until treatment acts: when it decreased, resistance increased (PRCC ∼0.6). Furthermore, the multiresistance rate was positively sensitive to PCV7 use and negatively to the frequencies of antibiotic exposure in all age groups. Transmission rates between and within age-classes did not significantly influence the global resistance rates.

**Table 2:** Sensitivity analysis of the frequency of ketolide-resistant or multiresistant strains in the colonized population.

Parameter	Variable	PRCC^a^
Frequency of ketolide-resistant strains as output		
Frequency of ketolide exposure in adults (>15 years)	*α*_33_	0.3
(Duration of antibiotic exposure)^−1^	*γ*	−0.7
(Time until treatment acts)^−1^	*ν*	0.6
Probability of S→R incremental resistance of macrolide/ketolide	*p*_4_	0.22
Frequency of multiresistant strains as output		
Frequency of exposure		
*β*-Lactam in children (2–15 years)	*α*_12_	0.1
*β*-Lactam in adults (>15 years)	*α*_13_	0.2
Ketolide in young children (<2 years)	*α*_31_	0.1
Macrolide in adults (>15 years)	*α*_23_	0.1
(Duration of antibiotic exposure)^−1^	*γ*	−0.6
(Duration of colonization in adults)^−1^	*λ*_3_	0.1
(Time until treatment acts)^−1^	*ν*	0.6
Vaccination rate	*v*	−0.2

a Partial rank correlation coefficient (PRCC) indicates the degree of monotonicity between a specific input variable and a particular outcome variable. The sign of the PRCC indicates the qualitative relationship between input and output variables. The magnitude indicates the importance of uncertainty in estimating the value of the input variable in contributing to the imprecision in predicting the value of the outcome variable.

## Discussion

The results of this simulation study suggest that new antimicrobial use could markedly slow the diffusion of (I_P_/R_P_)+R_M_ multiresistant strains. During the initial period after ketolide introduction, their wider use was associated with a slower progression of multiresistance. The model predicted it would take >15 years to reach 10% of R_K_ in the population when the new drug was prescribed to all ages, and >40 years, when it was prescribed only to adults. In all cases, except when ketolides replaced all other drugs in the community, bacterial resistance increased, eventually reaching high rates of strains multiresistant to the 3 drugs [(I_P_/R_P_)+R_M_+R_K_], due to co-selection of R_M_+R_K_ strains under ketolide exposure. When all drugs but ketolides were discontinued, the resistance to *β*-lactams was contained at ∼70%. Obviously, this unrealistic strategy raises contradictory economic questions: should new drugs be developed in order to preserve old drug efficacy?

Current trends in *Streptococcus pneumoniae* epidemiology are difficult to anticipate: first, the epidemiological situation is complex because of multiresistant strains and multi-exposures to antibiotics; second, important changes have occurred recently with the availability and wide use of PCV7 and the measures undertaken to reduce antibiotic prescriptions. In the past, models helped to analyze probable changes, predicting changes in serotypes after vaccine use [23], [39], investigating the associations between antibiotic use and R_P_ [40] or explaining why *β*-lactam resistance presented a bimodal shape [41]. Here, using a model taking these recent changes into account, we investigated how the use of a new antimicrobial can modify the resistance patterns in the community.

As non-vaccine strains are currently less resistant to macrolides [9], [42], immunization may delay the occurrence of R_K_. For that reason, and also because wide vaccine coverage might select for non-vaccine–serotype multiresistant stains [10], PCV7 was included in this study. No precise estimate exists for the duration of PCV7-induced immunity as the vaccination program started only a few years ago. In our model, we assumed that PCV7-induced immunity would last throughout childhood. A shorter (longer) duration of immunity would be nearly equivalent to predictions of immunizing a smaller (greater) portion of young children.

The model and results presented here are specific to the introduction of ketolides into a population exposed to high rates of penicillin and macrolide use.

First, the study was restricted to penicillin, macrolides and ketolides, excluding other antibiotics, such as quinolones or cyclines. In most countries, as in France, *β*-lactams and macrolides are the most frequently prescribed antimicrobial classes. Moreover, *S. pneumoniae* resistance to them has reached high percentages in several Western countries and become a major public health concern [11]. In contrast, fluoroquinolone and cycline antibiotic resistances remain a quite low [35] and are not, at this time, a worrisome problem in terms of clinical failure in Europe and the US. For those reasons, we focused on the control of R_P_ and R_M_ and multiresistance. Considering other antibiotics and *S. pneumoniae* resistance to them in the model would have had little to no effect on the predicted changes in the main resistance patterns in the community in the near future.

Second, we described the pathway to R_K_ based on current observations: R_K_ can be developed only in R_M_ strains. This assumption effectively limits the selection for R_K_ to strains already R_M_, so that progression to resistance is slower than in a setting in which resistance to the new drug would appear independently of others. The evolution of resistance to such a drug could easily be analyzed in the same setting: it is expected that, in the same context, the emergence of a new resistance would be faster but the selection slower.

Third, the efflux-pump resistance mechanism, which endows *S. pneumoniae* with low-level R_M_, was not considered in the model. Indeed, for macrolides, the model developed herein was based on the French situation. In France, the vast majority of R_M_ isolates have acquired a ribosomal modification resistance mechanism [35]. Moreover, the efflux-pump mechanism does not impair ketolide activity, while the ribosomal modification mechanism has marked impact in terms of selective pressure on both R_M_ and R_K_. Consequently, in the French context, adding the possibility of 3 different levels of macrolide-susceptibility (susceptible, low-level resistant, and highly resistant) would have limited influence on the predictions.

Fourth, the model could also be used in a setting with lower exposure to penicillins and macrolides. In that case, initial R_P_ and R_M_ rates would also be lower, and the emergence and selection of new resistances would be expected to slow, and the impact on multiresistance trends of a new antibiotic to be delayed.

In the scenarios, we assumed a sudden replacement of part of current antibiotics by the innovative agent, while it could be more progressive over time in real life. The simulated situation constitutes a worst-case scenario for the selection of R_K_. Therefore, R_K_ emergence would most likely be delayed in practice, but at most by the length of the progressive replacement period. Likewise all other kinetic steps would occur later.

The model uses several simplifications regarding the diversity of *S. pneumoniae* ecology and the results presented should be interpreted in the context of the following 3 limitations. First, our simulation did not examine the time required for pneumococci to acquire a new R_K_ mechanism, but rather that needed to reach an epidemiologically meaningful resistance level in the population. At present, few R_K_ strains exist in the community [37]; therefore, we focused here on the time needed to select and spread these resistant strains depending on the different drug-use scenarios (figure 4). Second, the possibility of colonization (and transmission) with several strains having different resistance patterns was not considered in this study. Lastly, serotypes were grouped according to their inclusion in the PCV7 rather than individually modeled. A higher level of details could be achieved, for instance using serotype-specific duration of carriage or immunity to *S. pneumoniae* [43]. However, the main conclusions regarding the impact of ketolides on the emergence and progression of multiresistance would not be affected by these latter choices.

In our sensitivity analysis, the parameters that caused the largest changes in results were: the frequencies and duration of antibiotic exposure (when they increased, the proportion of resistant strains increased); and the mean time until treatment acts (when was shorter, the mean time for resistance selection took longer and resistance became more prevalent). When antibiotics eliminate sensitive strains more quickly, the opportunity for sensitive strains to become resistant is decreased; however, once resistance has emerged, resistant strains have a stronger selective advantage.

It has been suggested [44], [45] that the transmissibility of resistant strains could be lower than that of susceptible strains because resistance acquisition could imply a fitness cost. We did not include any fitness difference in our model. In terms of prediction, we would expect that introducing fitness cost for resistant strains, which increases with multiresistance, would delay the second-wind rise of multiresistance in non-vaccine strains. To date, however, biological evidence on this possibility at the human level is sorely lacking [46].

Recently, the US Food and Drug Administration approved a new label for ketolides, removing specific indications (acute bacterial sinusitis and acute exacerbation of chronic bronchitis) and adding warnings and contraindications [47]. This new labeling was incited by a rare hepatitis linked to ketolide use [48], [49]. Consequently, the ketolide-use benefit predicted by the model should be balanced by the incidence of severe adverse effects. At this point, precise quantification of the costs, morbidity and mortality associated with pneumococcal antibiotic resistance becomes urgent [50]–[52]. Modeling, complemented with such data, should enable estimation of benefits and risks and should become a real support for public health decision-making.

Today, the only available ketolide drug, telithromycin, is approved for use only in adults, although children represent a large portion of antibiotic users. However, other new ketolide-class antibiotics, with the same activity as telithromycin, like cethromycin, are being developed [53], [54] and could be prescribed to children in the future. In that context, the model predictions could show how the use of such an antibiotic innovation could impact the global pattern of resistance, at the population level.

We want to underline that the main pathways for resistance control remain the global reduction of antibiotic consumption, generalized PCV7 immunization of young children, and cautious and appropriate use of antibiotics at the individual level. While the results of this study emphasize the potential benefits of a partial replacement of currently used antibiotics by a new antibiotic class, our conclusion is not to encourage the wide use of ketolides but to demonstrate that innovation could be a complementary way to reduce antibacterial resistance at the community level.

According to our model, restriction of ketolide prescriptions to adults, even at a high replacement rate, would have a limited effect on the global resistance pattern and would mainly lead to clinical success. When extended to children, ketolide use would be associated with only a short-term positive effect. Using a model that included age-structured population and specific resistance and multiresistance mechanisms (cross-resistance and co-resistances), our findings are in agreement with the situation described by Wang and Lipsitch [55]: upgrading antibiotic use within a class (fluoroquinolones vs macrolides/ketolides here) would lead to a trade-off between high prevalence of highly multiresistant strains and successful treatment. Facing the choice between enhancing therapeutic success and controlling global resistance, these models can be useful for decision-makers.

In conclusion, our simulation results predict that, in a population with widespread use of PCV7 and rational use of antibiotics, antibiotic innovation, prescribed to all age groups, particularly children, could have an added impact on multiresistance rates: the more the new drug is prescribed, the slower multiresistance would diffuse. However, at the same time, the more this new drug is used, the faster new multiresistant pneumococcal strains ((I_P_/R_P_)+R_M_+R_K_ strains here) would be selected.

Paradoxical as it may seem, to prevent community-wide dissemination of strains simultaneously resistant to all available antibiotics, the optimal solution for the next 20-years, would be to switch antibiotics totally, preserving one or more of the older drugs for which non-resistant strains still exist.
