# Correction: Antibiotic Innovation May Contribute to Slowing the Dissemination of Multiresistant *Streptococcus pneumoniae*: The Example of Ketolides

**DOI:** 10.1371/annotation/968c3453-14aa-4300-b743-cdb4f4c90a36

**Published:** 2008-05-08

**Authors:** Lulla Opatowski, Laura Temime, Emmanuelle Varon, Roland Leclercq, Henri Drugeon, Pierre-Yves Boëlle, Didier Guillemot

The fourth author's name appears incorrectly. It should be spelled: Roland Leclercq

